# Tannic acid modified keratin/sodium alginate/carboxymethyl chitosan biocomposite hydrogels with good mechanical properties and swelling behavior

**DOI:** 10.1038/s41598-024-63186-6

**Published:** 2024-06-04

**Authors:** Liqing Zhu, Fenfen Ouyang, Xue Fu, Yimei Wang, Ting Li, Min Wen, Guodong Zha, Xue Yang

**Affiliations:** 1 College of Chemistry and Chemical Engineering, Chongqing University of Science and Technology, Chongqing, China; 2 Department of Pharmacy, Army Medical Center of PLA, Chongqing, China; 3 HEMOS (Chongqing) Bioscience Co., Ltd., Building #2, No.216, Jianshan Road, Bishan District, Chongqing, China

**Keywords:** Hydrogels, Keratin, Tannic acid, Wound healing, Biomedical materials, Biomedical engineering

## Abstract

Natural polymer-based hydrogels have demonstrated great potential as wound-healing dressings. They help to maintain a moist wound environment as well as promote faster healing. In this work, a multifunctional hydrogel was prepared using keratin, sodium alginate, and carboxymethyl chitosan with tannic acid modification. Micro-morphology of hydrogels has been performed by scanning electron microscopy. Fourier Transform Infrared Spectroscopy reveals the presence of hydrogen bonding. The mechanical properties of the hydrogels were examined using a universal testing machine. Furthermore, we investigated several properties of the modified hydrogel. These properties include swelling rate, water retention, anti-freezing properties, antimicrobial and antioxidant properties, hemocompatibility evaluation and cell viability test in vitro. The modified hydrogel has a three-dimensional microporous structure, the swelling rate was 1541.7%, the elastic modulus was 589.74 kPa, the toughness was 211.74 kJ/m^3^, and the elongation at break was 75.39%, which was similar to the human skin modulus. The modified hydrogel also showed inhibition of *S. aureus* and *E. coli*, as well as a DPPH scavenging rate of 95%. In addition, the modified hydrogels have good biological characteristics. Based on these findings, the K/SA/CCS hydrogel holds promise for applications in biomedical engineering.

## Introduction

A wound dressing is an essential material that provides a temporary protective physical barrier and rapid wound healing^1^. The ideal wound dressing should have the following characteristics^2–4^: a moist environment, rapid wound healing, mechanical protection, no cytotoxicity to healthy tissues, antibacterial/antifungal action, and high patient compliance. As a hydrophilic matrix, hydrogel consists of a three-dimensional network of hydrophilic polymers which can provide the moisture necessary to optimize re-epithelialization, carrier-supporting effect on the drug, and automatically absorb wound drainage^5–7^. Keratin is a most abundant animal sourced biopolymer which is extracted from several sources including wool, human hair, chicken feathers and many others^8^. Keratin exhibits good bioactivity, biocompatibility, biodegradability, and low immuno-exclusion that can provide new alternatives for designing novel biomaterials^9–11^. Moreover, the Leu-Asp-Val (LDV), Glup-Asp-Ser (EDS), and Arg-Gly-Asp (RGD) cellular sequences of keratins favor cell adhesion and proliferation^11,12^. Therefore, it has potential advantages in the field of regenerative medicine. For example, bioscaffolds^13,14^, films^15^, hydrogels^16^, adsorbents^17^, additives^18^, and spinning slurries^19^ using keratin as a bio-based material are widely used in bone growth, hemostasis^20^, wound repair^21^, and water treatment^22^. However, the utilization of pure keratins in promoting wound healing has been limited due to their high brittleness, poor toughness, and insufficient mechanical properties^23^.

Sodium alginate (SA) is a highly promising biopolymer. SA is a linear unbranched, amorphous copolymer composed of α-L-guluronic acid (G) and 1,4-linked β-D-mannuronic acid (M) residues^24^. It is a water-soluble, naturally occurring polysaccharide derived from brown algae and seaweeds that provides excellent properties such as good film formation, hydrophilicity, chelating, biocompatibility, non-toxicity, and relatively low-cost^25,26^. The current study confirms that SA can be used to improve the mechanical properties of biomaterial. Carboxymethyl chitosan (CCS) is a water-soluble chitosan derivative with better biocompatibility, biodegradability, antimicrobial activity, and moisturizing ability^27^. In recent years, CCS and SA polysaccharide-based hydrogels induced by electrostatic interactions have attracted much attention due to their excellent water absorption and biocompatibility^28,29^. Their incredible mechanical strength and toughness can be achieved by tuning the inter-/intramolecular interactions through monomers, cross-linking agents, and synthesis methods. Muhammad et al. have attempted to prepare CCS/SA physic hydrogels using a Genipin green cross-linking agent^30^. Their report indicates that although the mechanical properties of the hydrogel were improved, the swelling behavior of the hydrogel was severely sacrificed, and maintaining a balance between the swelling properties and the mechanical properties of the hydrogel remains a challenge.

Chen et al. improved feather keratin/gelatin nanofiber nonwoven fabrics by glutaraldehyde cross-linking, which was made possible by electrostatic spinning to cross-link keratin in glutaraldehyde vapor, effectively improving its mechanical strength and water resistance. Yet, due to the toxicity of glutaraldehyde, the biocompatibility of keratin is limited^31^. Tannic acid (TA) is a natural plant-derived polyphenol molecule formed by the ester bond between the five hydroxyl groups of a glucose core and the gallic acid group. The protein modification, surface functionalization, or substance interaction through molecular complexation, cross-linking, and various non-covalent interactions have been reported involved in TA literature^32^. Moreover, TA is biocompatible with antioxidant, antimicrobial, and anti-inflammatory properties, consequently, it is a non-toxic cross-linking agent that can be used in the preparation and modification of biomaterials^33^.

In this study, hydrogels were designed and prepared using tannic acid for green crosslinking modification. The gel matrix consisted of keratin, sodium alginate, and carboxymethyl chitosan natural polysaccharides. Keratin was chosen for its ability to promote cell adhesion and proliferation in wound healing, while sodium alginate and carboxymethyl chitosan were included to enhance the mechanical properties of the hydrogel. Fourier transform infrared spectroscopy (FT-IR) was used to characterize the chemical structure of multifunctional composite hydrogel. Scanning electron microscopy (SEM) was used to observe the micro-morphology and structure of multifunctional composite hydrogel. The mechanical properties of hydrogels have been measured by using a universal testing machine. Additionally, the hydrogels underwent testing for antimicrobial and antioxidant properties, water retention, anti-freezing properties, blood compatibility and cell viability test in vitro.

## Results and discussion

### FT-IR

**Figure 1:**
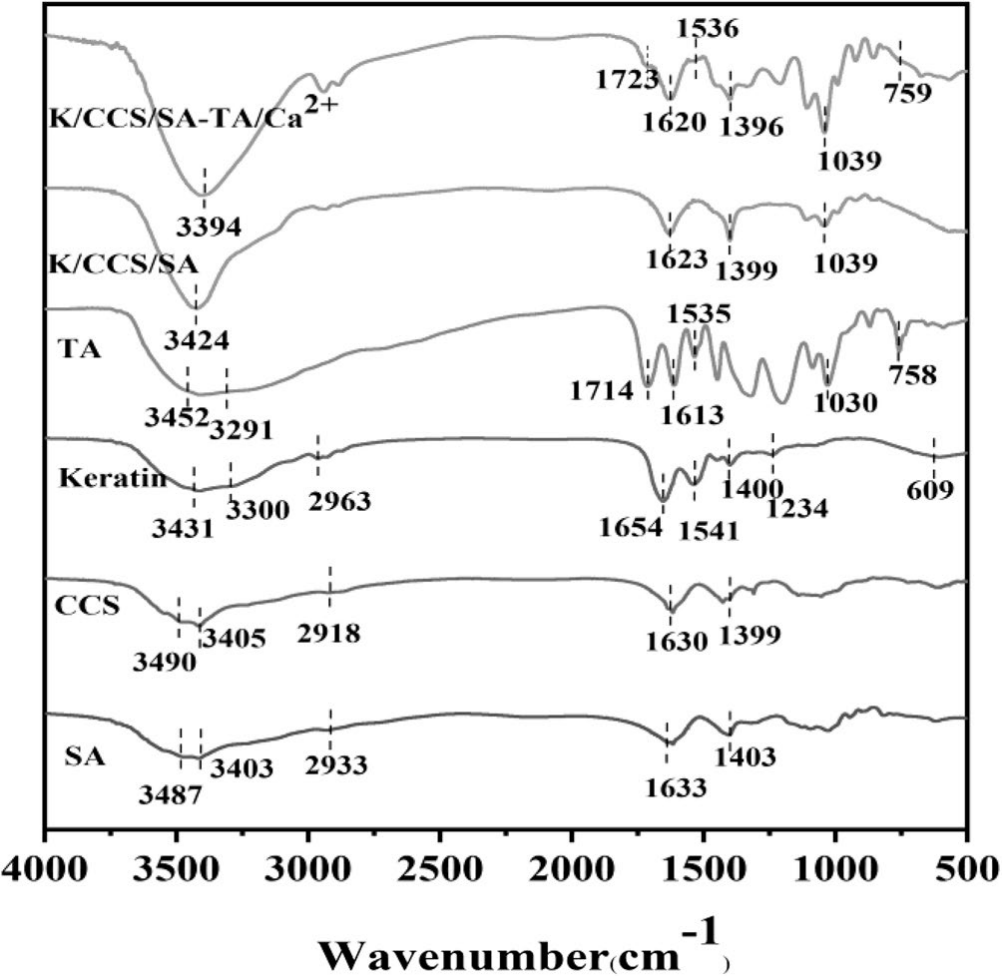
FT-IR spectra of the SA, CCS, Keratin, TA, K/CCS/SA, K/CCS/SA-TA/Ca^2+^.

As shown in Fig. 1, the FT-IR spectra of SA supported its structure by the appearance of the –OH band at 3487 cm^−1^, symmetric and asymmetric stretching vibrations of –COO groups at 1403 cm^−1^ and 1633 cm^−1^, respectively. The characteristic absorptions of CCS appeared at 3490 cm^−1^ of –OH and –NH_2_ stretching vibration absorption, symmetric and asymmetric stretching vibrations of –COO groups at 1399 cm^−1^ and 1630 cm^−1^, respectively. The stretching vibration absorption peaks of C-H groups appeared at 2918 cm^−1^. The FT-IR spectrum of keratin indicates several critical peaks and their corresponding vibrations. The similar typical characteristic at 3405 cm^−1^ corresponds to t the stretching vibration of –OH and –NH_2_ groups. The stretching vibrations of the C=O stretching (amide I), N–H stretching (amide II) and C–N bending (amide III) were also found at 1654 cm^−1^, 1541 cm^−1^ and 1234 cm^−1^, respectively, which confirmed that amide bonds exist in the keratin. The band observed at 2963 cm^−1^ corresponds to the stretching vibration of C–H bonds. Additionally, a distinctive absorption peak at 609 cm^−1^ indicates the presence of S–S groups. The spectra of the TA band at 3452 cm^−1^ hydroxyl and carbonyl broad stretching vibrational peaks and 1714 cm^−1^ the carbonyl stretching vibrational peak. The hydroxyl and amino peaks of the K/CCS/SA hydrogel were shifted to 3394 cm^−1^ after treatment with the tannic acid solution, and the carbonyl peaks may be due to the formation of hydrogen bonds between –NH_2_ and –OH in the hydrogel and –OH in the TA molecule. The carbonyl stretching vibrational peak of tannic acid has shifted from 1714 to 1723 cm^−1^, indicating that the carbonyl group's vibrational activation energy is affected by the carbonyl group's interaction with the hydrogen donor. In addition, the absorption peaks spectra of hydrogel mixed with TA at 1613 cm^−1^, 1535 cm^−1^, and 758 cm^−1^ attributed to the C=O, C=C, and C–H stretching vibrations of the substituted benzene ring, respectively, suggesting that TA has been successfully introduced into the K/CCS/SA matrix, which can stabilize the internal structure of the hydrogel.

### Mechanical properties: Effect of TA content on hydrogel tensile properties

**Figure 2:**
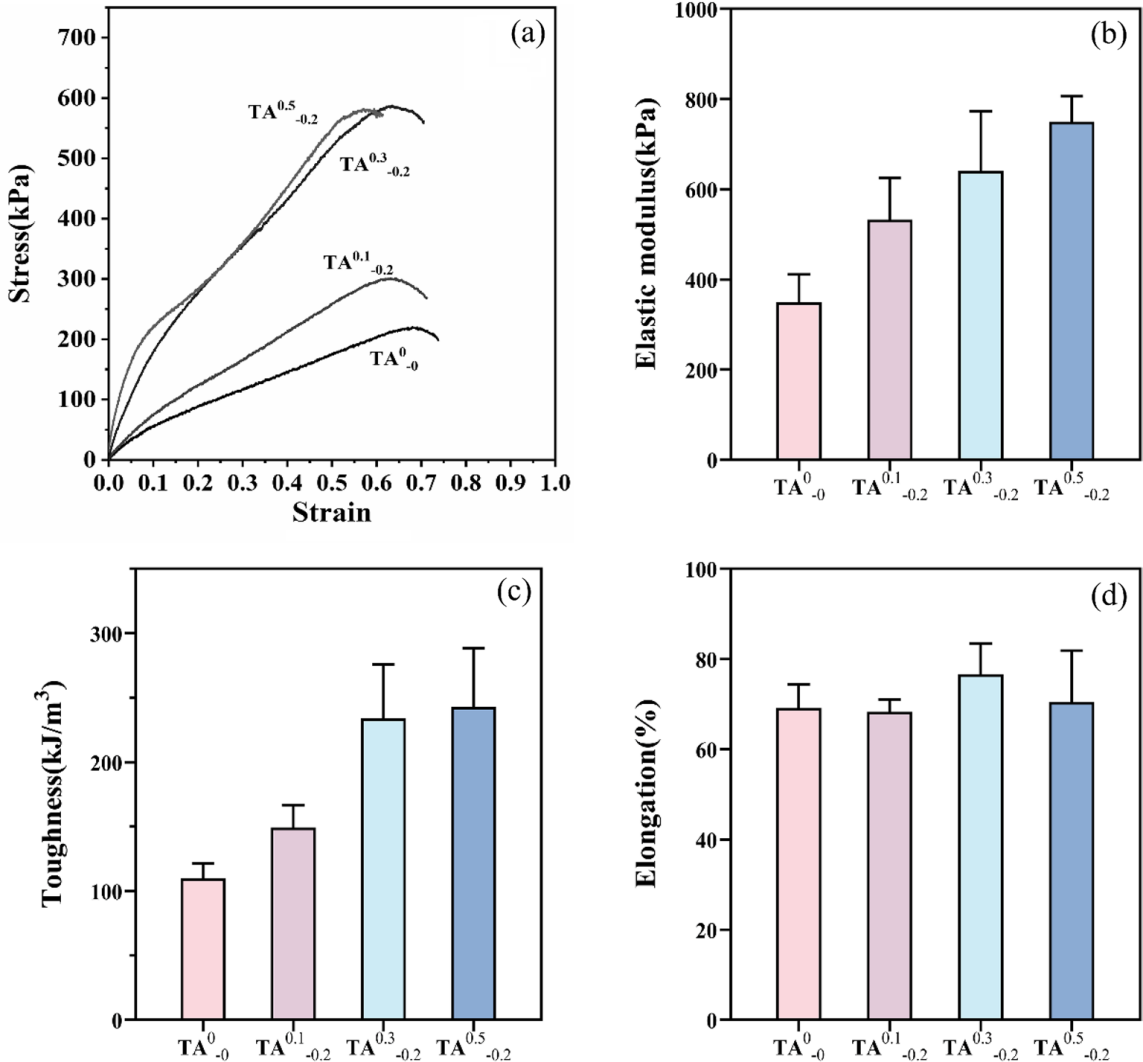
Mechanical properties of disparity TA hydrogels (**a**) Stress–strain curve, (**b**) Modulus of elasticity, (**c**) Toughness, (**d**) Elongation at break.

**Figure 3:**
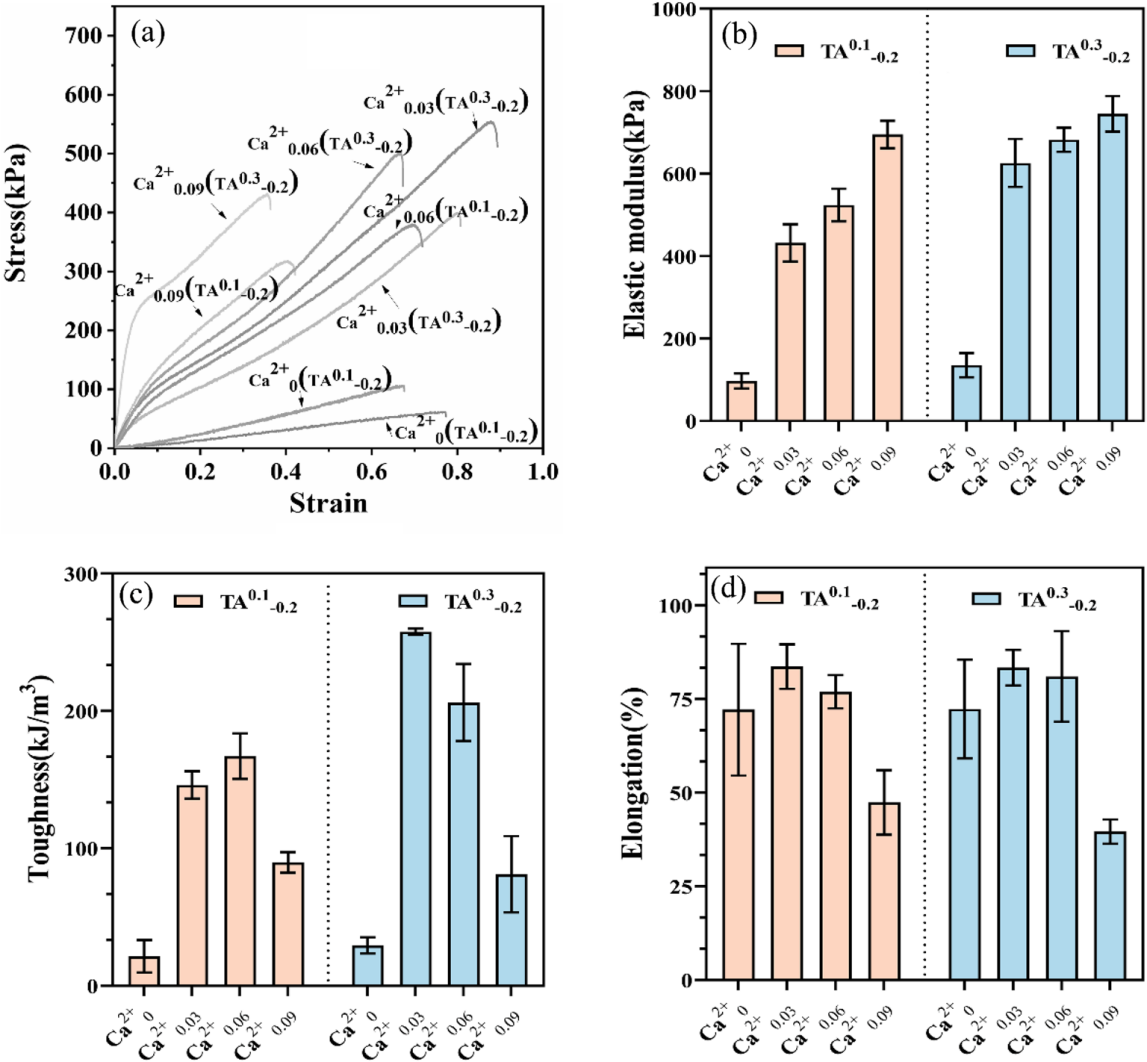
Mechanical properties of disparity Ca^2+^ hydrogels (**a**) Stress–strain curve, (**b**) Modulus of elasticity, (**c**) Toughness, (**d**) Elongation at break.

The excellent mechanical properties of hydrogels can facilitate the durability and stability of wound dressings during clinical application. A universal testing system measured the tensile properties of hydrogels with different TA contents. The mechanical stability of the fabricated hydrogels was elucidated by finding the elastic modulus, elongation, as well as toughness at break Fig. 2a shows the stress–strain curve while elastic modulus, toughness, and elongation percentage are depicted in Fig. 2b–d, respectively. After the introduction of an amount of TA, the elastic modulus of the hydrogels increased slightly from 349.348 kPa for TA^0^_−0_ to 749.848 kPa for TA^0.5^_−0.2_, which were mainly via the formation of hydrogen-bonding interactions between a large number of hydroxyl groups on the TA and amino, carboxyl, peptide bonding, and hydroxyl groups on the K/CCS/SA gel matrix, thus demonstrating excellent mechanical properties. After comparing with the hydrogel without TA, the toughness of K/CCS/SA hydrogel showed a slow enhancement trend accompanying an increase in TA concentration. The data of Fig. 3c and d indicated that due to hydrogen bonding interactions, energy has been dissipated to increase the toughness and elongation of the hydrogel at break. Whereafter, the movement of free movement of water molecules in the hydrogel has been inhibited by excessive cross-linking, resulting in a decrease in elongation at break. Hereby, TA 0.3 (w/v) was chosen for subsequent experiments.

### Effect of Ca^2+^ concentration on the tensile properties of hydrogels

SA is a natural linear anionic polysaccharide that interacts with Ca^2+^ to form an "eggshell" structure, which in turn forms a physical hydrogel. Besides, due to the abundance of –OH groups in TA, it can also form complexes with Ca^2+^^34,35^. The data in Fig. 3 shows that upon an increase in the elastic modulus of the hydrogel, the variation trend in toughness and elongation at break increases first and then decreases. The result exhibits that the stability of the internal structure of the hydrogel has been improved since the further cross-linking between Ca^2+^ and SA/TA. However, the higher cross-linking density may decrease its toughness and elongation at break subsequently. Moreover, via comparing the tensile properties of TA^0.1^_−0.2_ and TA^0.3^_−0.2_ hydrogels, it was found that the mechanical properties of the hydrogels increased with the increase of TA content and the different Ca^2+^ content, which was attributed to the complexation between TA and Ca^2+^. In short, from our aforementioned investigation, 0.06 w/v Ca^2+^ and 0.3 w/v TA were selected for subsequent experiments.

### Effect of glycerol content on water retention of hydrogels

**Figure 4:**
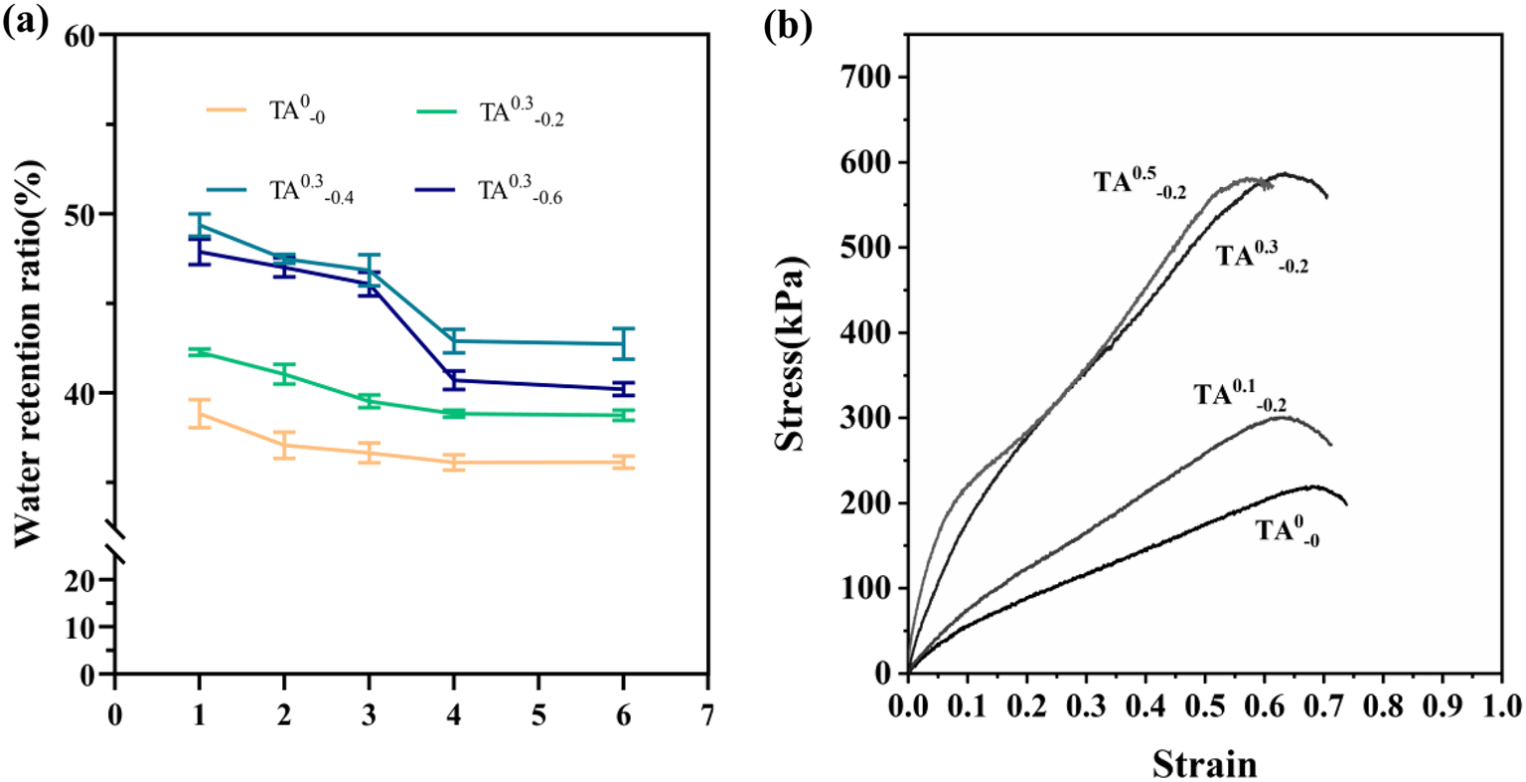
(**a**) Water retention graph of hydrogels with different glycerol contents (**b**) Stress–strain diagrams of TA^0.3^_−0.2_ and TA^0.3^_−0.4_ after 1d at 25 °C.

Glycerin can interact with water molecules in the hydrogel matrix to generate hydrogen bonds, which hinder the evaporation of water molecules, thus showing good water retention and frost resistance, maintaining the stability of the hydrogel and lasting effectiveness^36^. As shown in Fig. 4a, the water retention of the hydrogel increased and then decreased with the increase in glycerol content. From 1 to 6 days, TA^0.3^_−0.4_ can exhibit the eminent performance of water retention, even though it maintained the water retention at 42.89% until the sixth day. As in Fig. 4b, for comparison, under the same conditions, it can be seen that the modulus of elasticity of the TA^0.3^_−0.2_ hydrogel increased slightly after one day of placing the hydrogel at 25 °C, but the elongation at break decreased. In contrast, the elastic modulus of TA^0.3^_−0.4_ hydrogel increased, along with the elongation at break decreased.

**Figure 5:**
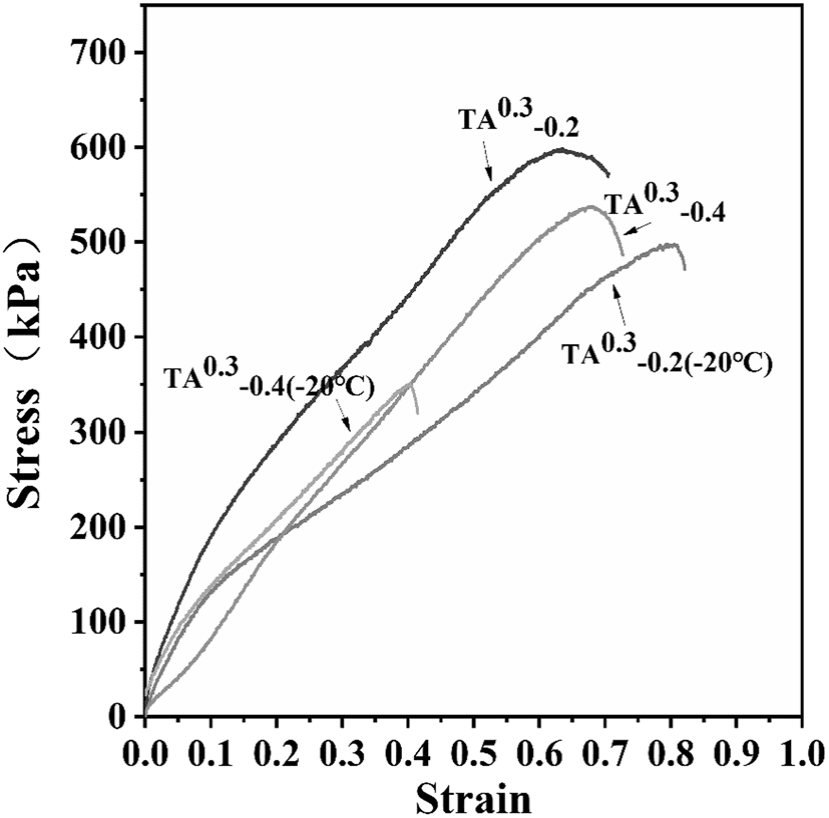
Stress–strain diagrams of TA^0.3^_−0.2_ and TA^0.3^_−0.4_ hydrogels after 1d at − 20 °C.

Conventional hydrogels are unable to maintain good mechanical properties at low temperatures due to icing, thus limiting the application of hydrogels in cold environments. In the presence of glycerol, the crystallization of water in the hydrogel can be effectively inhibited. As shown in Fig. 5, TA^0.3^_−0.2_ hydrogel and TA^0.3^_−0.4_ hydrogel were placed at − 20 °C, after 24 h, their elasticity modulus increased slightly, yet despite both hydrogels still maintained excellent flexibility, their elongation decreased at the break. In summary, TA 0.3 w/v, calcium ion concentration of 0.06 w/v, and glycerol content of 0.4 w/v hydrogels were selected for subsequent experiments.

In summary, TA 0.3 w/v, Ca^2+^ 0.06 w/v, and glycerol content of 0.4 w/v hydrogels were selected for subsequent experiments. In previous reports, the introduction of silver and graphene oxide in sodium alginate and chitosan gave a great improvement in the modulus of elasticity, but the effect of its elongation at break was poor^37^. Whereas in the present study, the TA^0.3^_−0.4_ hydrogel formed by the interaction of keratin, sodium alginate, and carboxymethyl chitosan through TA, had a modulus of elasticity of 589.74 kPa, a toughness of 211.74 kJ/m^3^, the elongation at break was 75.39%, and its mechanical properties matched those of human skin, and it had good water retention properties and maintained a good elastic modulus at low temperatures. Therefore, K/CCS/SA hydrogel can be used as a potential wound dressing.

### Rheological properties test

**Figure 6:**
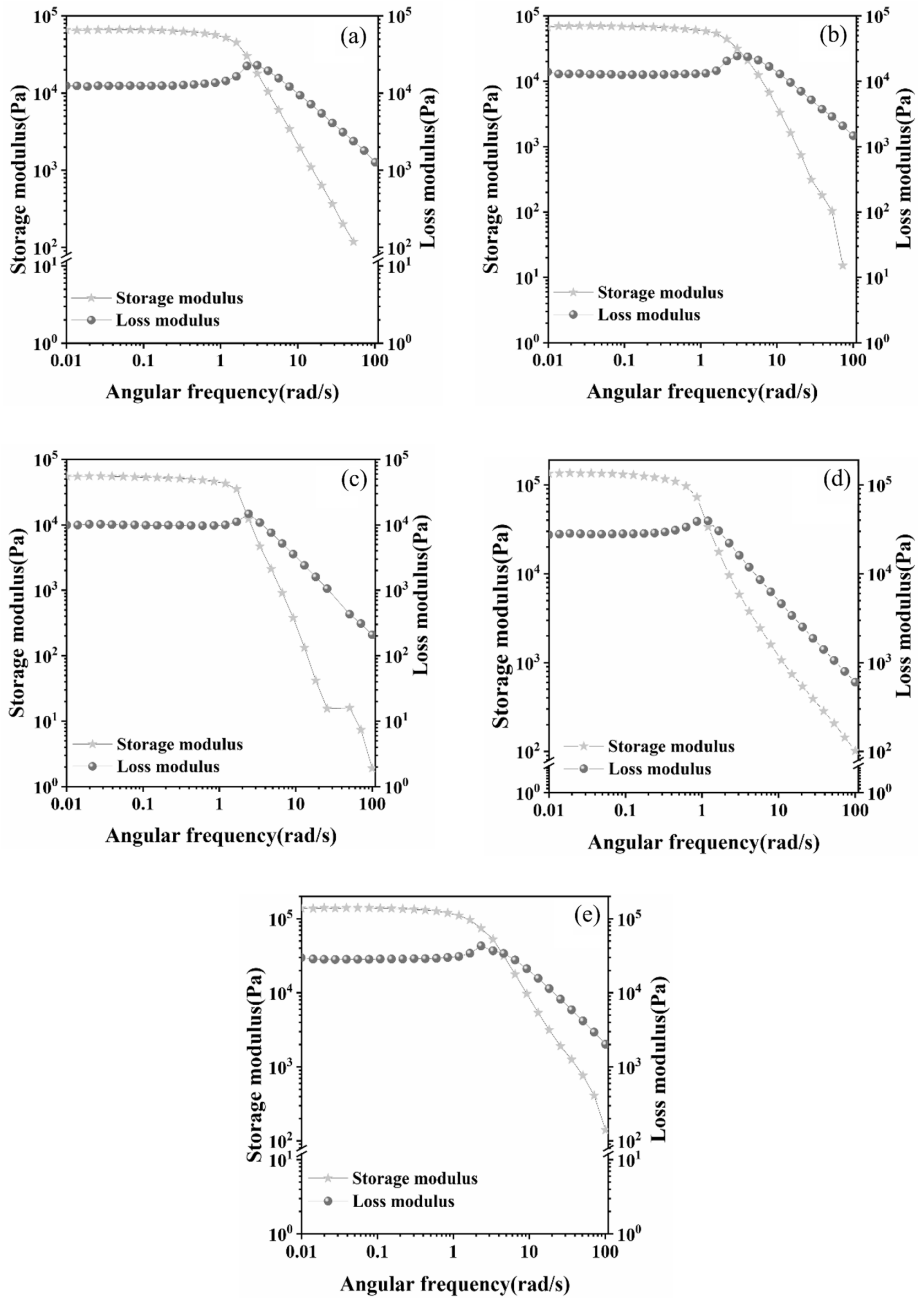
Energy storage modulus and loss modulus of different TA hydrogels (**a**) TA^0^_−0_ (**b**) TA^0.1^_−0.2_ (**c**) TA^0.3^_−0.2_ (**d**) TA^0.3^_−0.4_ (**e**) TA^0.5^_−0.2_.

**Figure 7:**
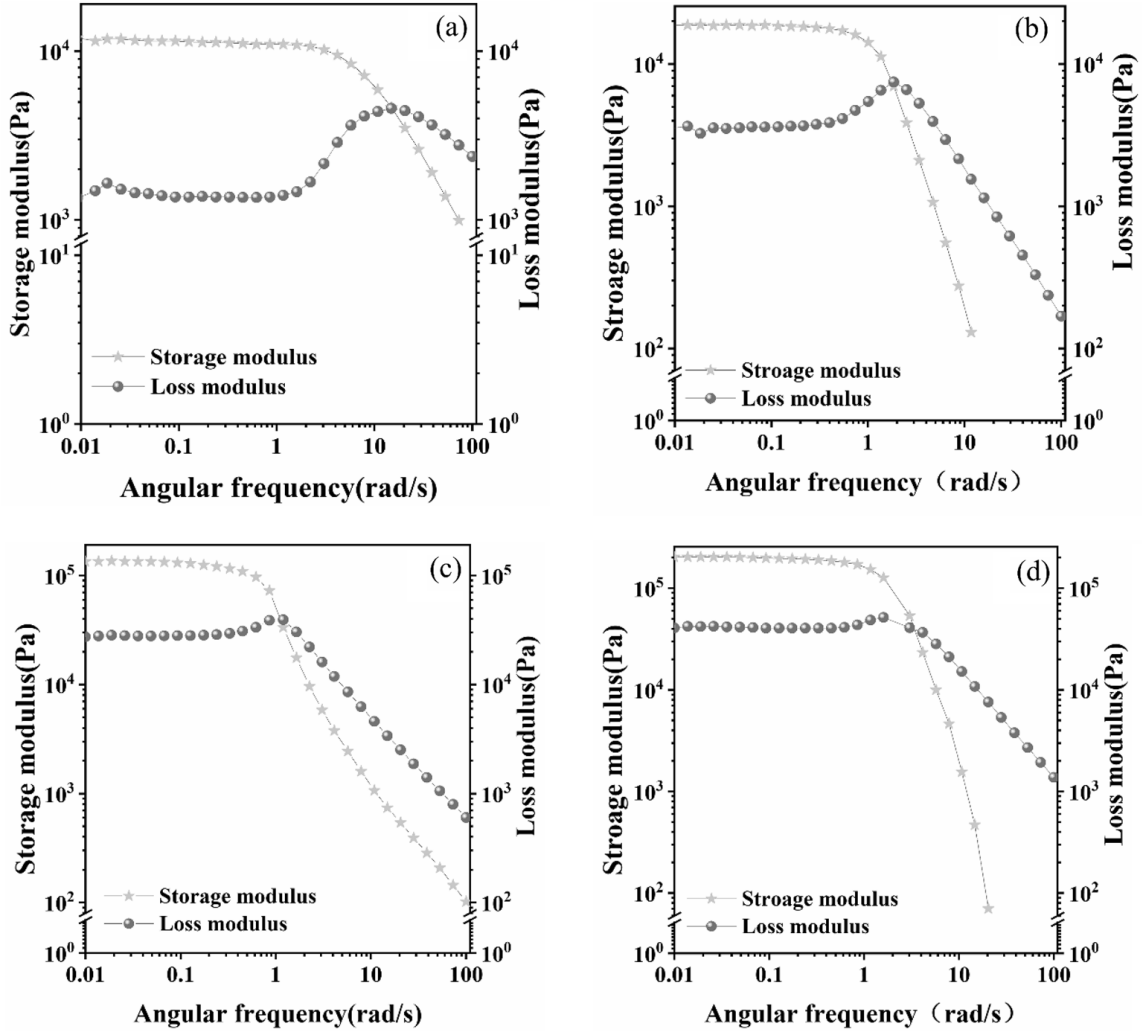
Energy storage modulus and loss modulus of different hydrogels for Ca^2+^ (**a**) Ca^2+^_−0_ (**b**) Ca^2+^_−0.03_ (**c**) Ca^2+^_−0.06_ (**d**) Ca^2+^_−0.09_.

In everyday life, individuals who use hydrogel dressings often experience various forms of pressure and deformation. Therefore, a rheological test was conducted to determine whether the hydrogel samples could maintain stability when subjected to different frequency scanning conditions. As depicted in Fig. 6, the initial state of the hydrogel is characterized by a significantly higher energy storage modulus (G') compared to the loss modulus (G"). This indicates that the hydrogel is in a solid-like state. As the scanning frequency increases, the intersection of G' and G" occurs, implying a transition from a solid-like state to a fluidic state for the hydrogel sample. Meanwhile, as the TA content increases, both G' and G" experience an increase. This increase in G' suggests improved elasticity of hydrogel, while the slight increase in G" indicates an elevation in the hydrogel's viscosity. It can be seen that the viscoelasticity and mechanical properties of the hydrogel will be improved substantially after the addition of TA. As shown in Fig. 7, at a specific concentration of TA^0.3^_−0.4_, both G' and G" exhibit an increasing trend with the rise in Ca^2+^ concentration. This observation suggests an enhancement in the viscoelasticity of the hydrogel.

### Swelling ability

**Figure 8:**
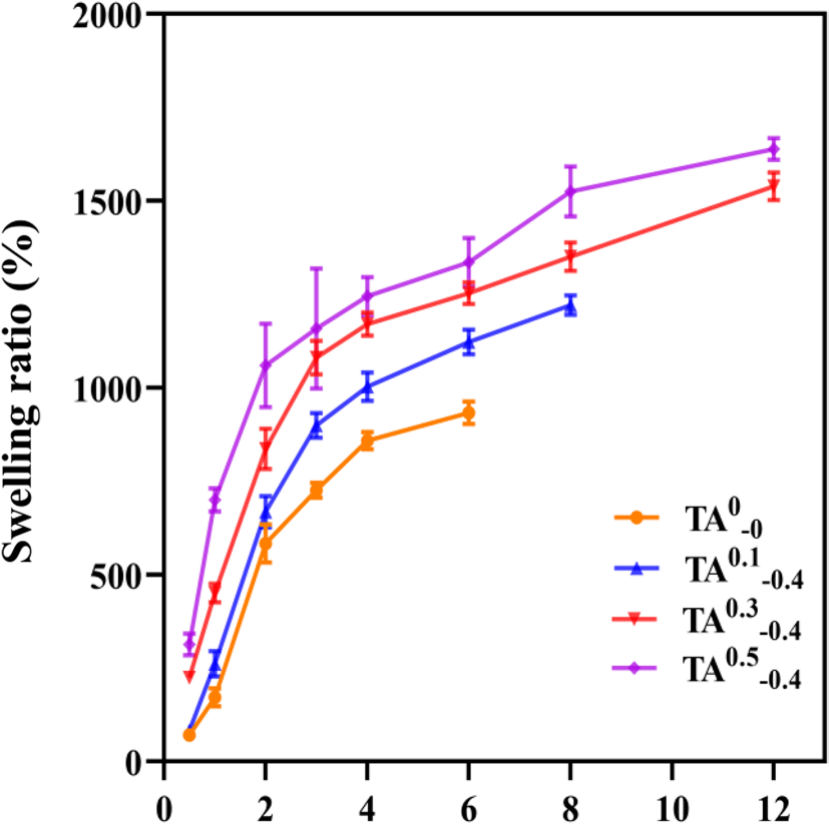
Swelling ratio of hydrogels with different contents of TA.

The swelling property of hydrogel indicates that it has good water adsorption ability, which, on the one hand, is conducive to the absorption of exudate and bleeding from the wound site and accelerates skin healing. On the other hand, the appropriate swelling property can maintain the moistness of the wound site, which is conducive to the maintenance of a typical physiological environment and contributes to the formation of epithelialization, thus contributing to the healing of the damaged tissues^38^. Therefore, the lyophilized K/CCS/SA hydrogels modified with different tannic acid solubility were placed in PBS solution at 37 °C, and their swelling behaviors are shown in Fig. 8. The swelling rate of all hydrogel samples increased rapidly within the first 4 h, followed by a more gradual increase. Immersion in PBS solution for 6 h, TA^0^_−0_ reached equilibrium with a swelling rate of 936.67%, while TA^0.1^_−0.4_ hydrogel took 8 h to get equilibrium with a swelling rate of 1121.7%. In comparison, TA^0.3^_−0.4_ and TA^0.5^_−0.4_ hydrogels reached equilibrium at 12 h with swelling rates of 1541.7% and 1660.0%, respectively. Moreover, the swelling ratio of hydrogels increased with growing TA content. This swelling behavior might be attributed to the presence of numerous phenolic hydroxyl groups in TA, which enhances the hydrophilicity and solubility properties of the hydrogel samples. Previously, it was reported that Kakkar's keratin-silica hydrogel prepared by tetraethyl orthosilicate reached saturation at 4 h, with an average swelling rate of 540%^39^. The swelling rate of TA^0.3^_−0.4_ keratin complex hydrogel prepared in this study was 2.85 times higher than that of TA^0.3^_−0.4_ keratin complex hydrogel. The higher swelling rate could keep the wound moist and help absorb the wound exudate, thus accelerating wound healing.

### SEM

**Figure 9:**
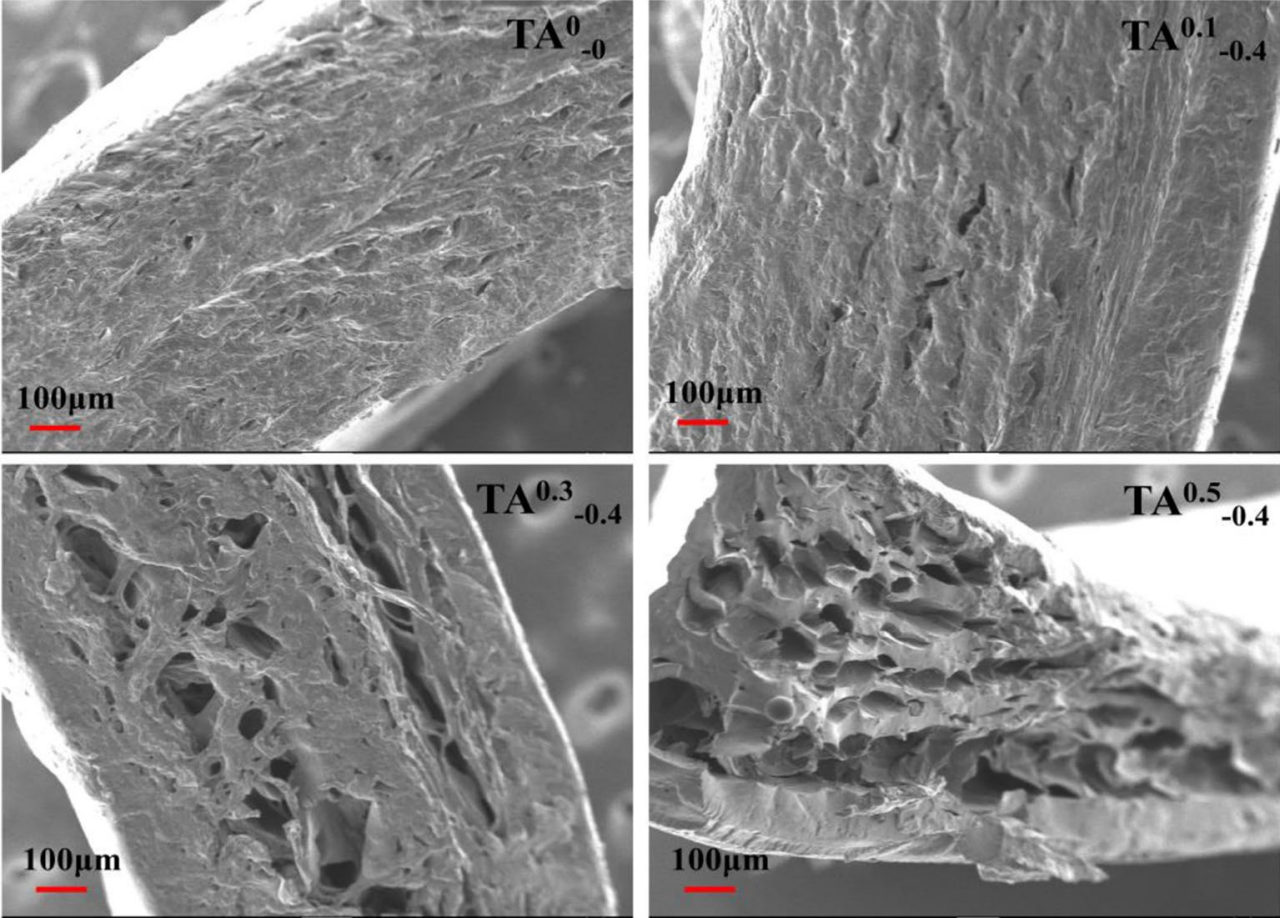
SEM images of hydrogels with different contents.

**Figure 10:**
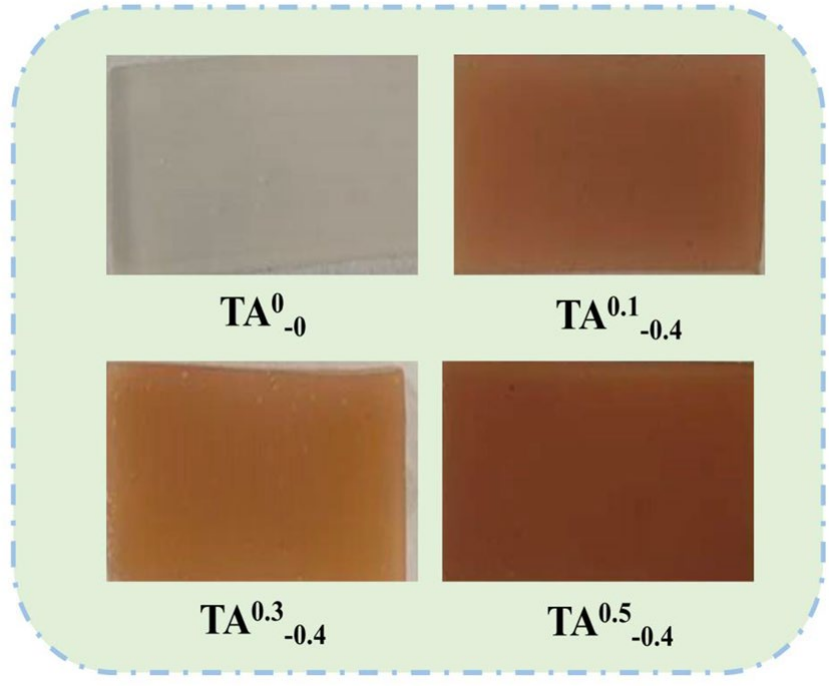
Physical diagram of hydrogel.

The SEM images of the four hydrogel samples are shown in Fig. 9, while Fig. 10 is a physical image of the hydrogel. It can be observed the cross-section surface structure of the TA^0^_−0_ sample has a higher density than TA^0.1^_−0.4_, TA^0.3^_−0.4_ and TA^0.5^_−0.4_ in Fig. 9. Similar interconnected porous structures appeared in the cross-section of the TA^0.3^_−0.4_ and TA^0.5^_−0.4_ samples with the increase in TA content. This is considered as the large number of phenolic hydroxyl groups on the TA benzene ring, which are prone to produce overlapping electron clouds. Also, the phenolic hydroxyl groups carry progressively more O^−^ than –NH^3+^ in the K/CCS/SA gel matrix and the repulsion produced by the net negative charge of the O^−^, all of which may lead to the emergence of a porous structure, consequently, affect the biological properties of the substance. Thus, one can further demonstrate that the increase in TA content resulted in better-swelling properties of the hydrogels. Additionally, bacteriostatic properties are greatly facilitated by the presence of pore structures that significantly increase the specific surface area of the material, resulting in improved bacteriostatic efficiency.

### Antibacterial studies

**Figure 11:**
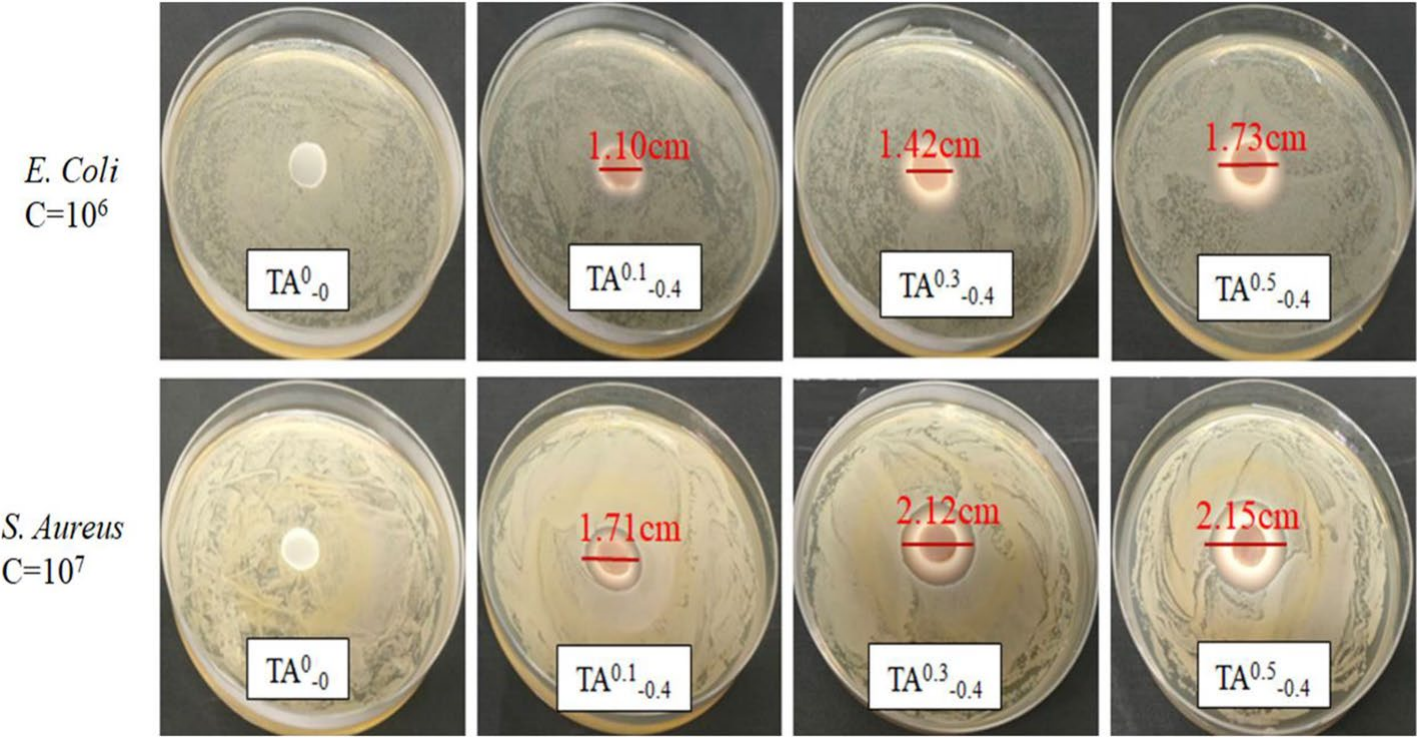
Inhibition of *Escherichia coli* and *Staphylococcus aureus* by hydrogels with different contents of TA.

**Figure 12:**
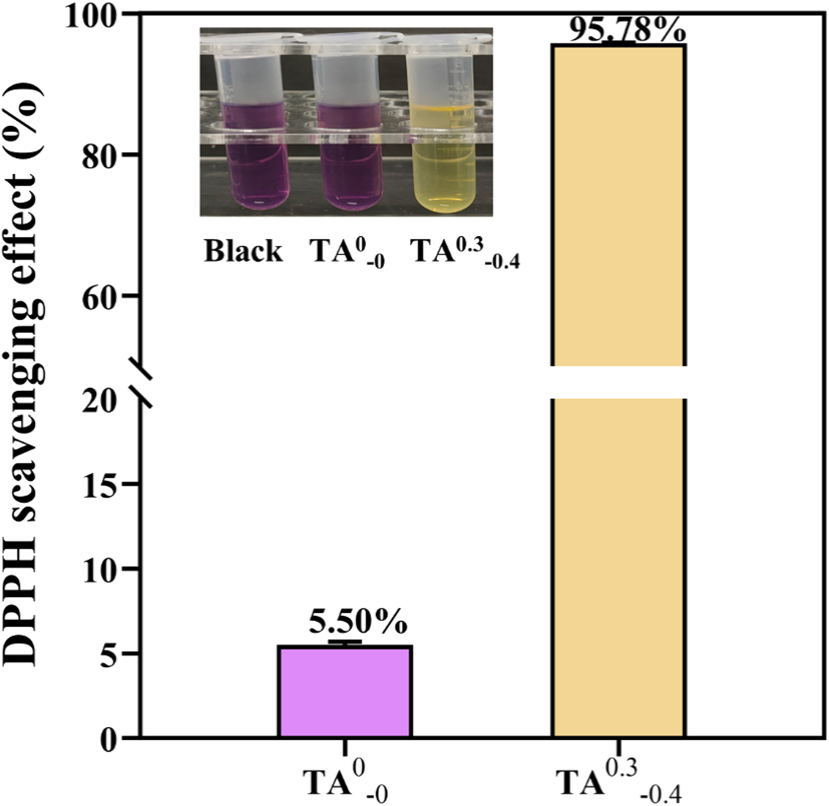
TA^0^_−0_ and TA^0.3^_−0.4_ hydrogels DPPH scavenging effect.

TA not only destroys the integrity of cell membranes through hydrogen bonding but also disrupts the membrane potential of bacteria, thereby inhibiting growth and reproduction^33^. TA can inhibit the activity of some important enzymes in bacteria, thus affecting their metabolic process and hindering their growth and reproduction. It can also bind to the proteins in bacteria and change the conformation and function of the proteins, thus inhibiting growth and reproduction. Figure 11 displays the diameter of the zone of inhibition for the hydrogels obtained through the disk diffusion method. The hydrogels exhibited the zone of inhibition against both bacterial strains by releasing TA on the cellular membranes thereby killing bacterial cells. However, in Fig. 12, it indicates a bigger zone of activity against *E. coli* as compared to *S. aureus*. Since reducing the concentration of *E. coli*, the obvious inhibition zone (1.42 cm) appeared around the TA^0.3^_−0.4_ hydrogels. The antimicrobial effectiveness of the Ta^x^_−0.4_ hydrogel increases progressively with higher TA content.

### DPPH assay

It has been confirmed that induction of antioxidant activity in wound dressings has a positive effect on wound healing process, since antioxidants can regulate the overproduction of reactive oxygen species (ROS) and consequently prevent the undesirable effect of these species^40^. To assess the antioxidant properties of the hydrogel dressings, Fig. 12 gives the result of their ability to scavenge the stable DPPH free radicals by monitoring the intensity of the DPPH radical absorption peak at 517 nm. The free extreme scavenging rate was 5.50% for TA^0^_−0_ hydrogel and 95.78% for TA^0.3^_−0.4_ hydrogel. Due to the radical scavenging capability of TA^0.3^_−0.4_ hydrogels, playing an equally important role was anticipated from designed wound dressing membranes.

### Hemolysis evaluation

**Figure 13:**
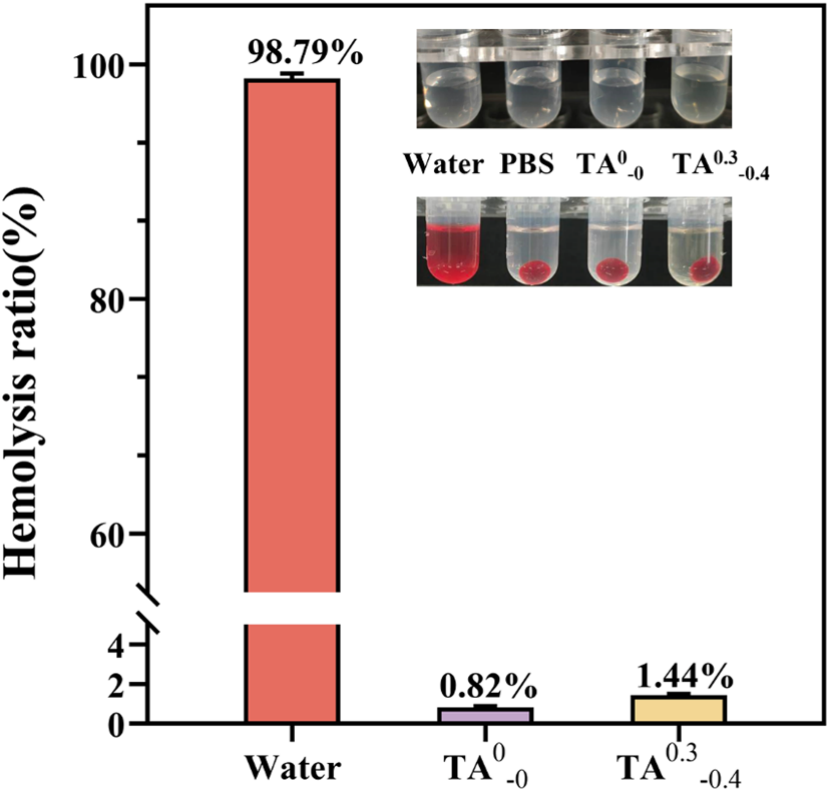
TA^0^_−0_ and TA^0.3^_−0.4_ hydrogels hemolysis ratio.

In the absence of skin protection, wound dressings can come into direct contact with subcutaneous tissue, so good blood compatibility is an essential attribute of wound dressings. As shown in Fig. 13, the positive control (water) was bright red, while the negative control and hydrogel sample groups had no significant color after incubation. The hemolysis rate was 0.82% for TA^0^_−0_ hydrogel and 1.43% for TA^0.3^_−0.4_ hydrogel. It indicates that the hydrogel sample meets the safety requirement of hemolysis rate < 5%, does not harm the cell membrane, does not lead to cell rupture, and can have excellent blood compatibility while taking into account the high bacteriostatic properties.

### Cell viability test in vitro

**Figure 14:**
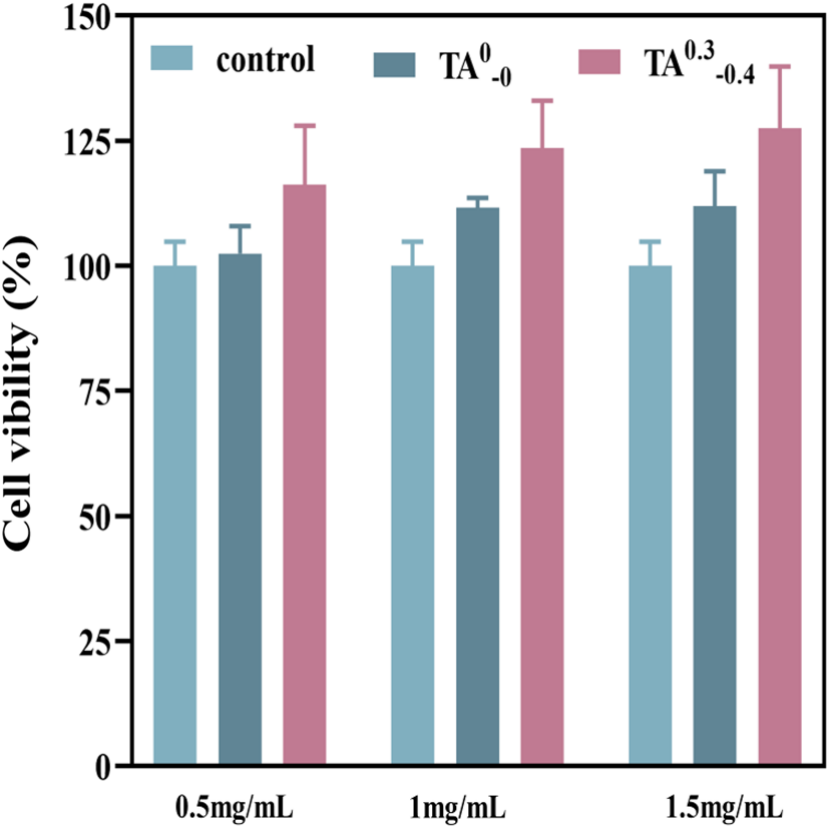
Cell viability of hydrogels at different concentrations.

The in vitro cytotoxicity assay has the advantages of simplicity, reproducibility, cost-effectiveness, and suitability for evaluating fundamental biological aspects of biocompatibility. In this experiment, the growth and proliferation of cells in hydrogel samples of different concentrations were investigated via the MTT method. As shown in Fig. 14, the cell viability after hydrogel extract treatment was greater than that of the control group in all cases, and the TA^0.3^_−0.4_ hydrogel was more effective than the TA^0^_−0_ hydrogel for cell proliferation; moreover, the cell viability increased with the increase in the concentration of hydrogel extract. Then, the experimental results showed that the addition of TA favored cell proliferation. In summary, the hydrogel dressing has good cytocompatibility.

**Figure 15:**
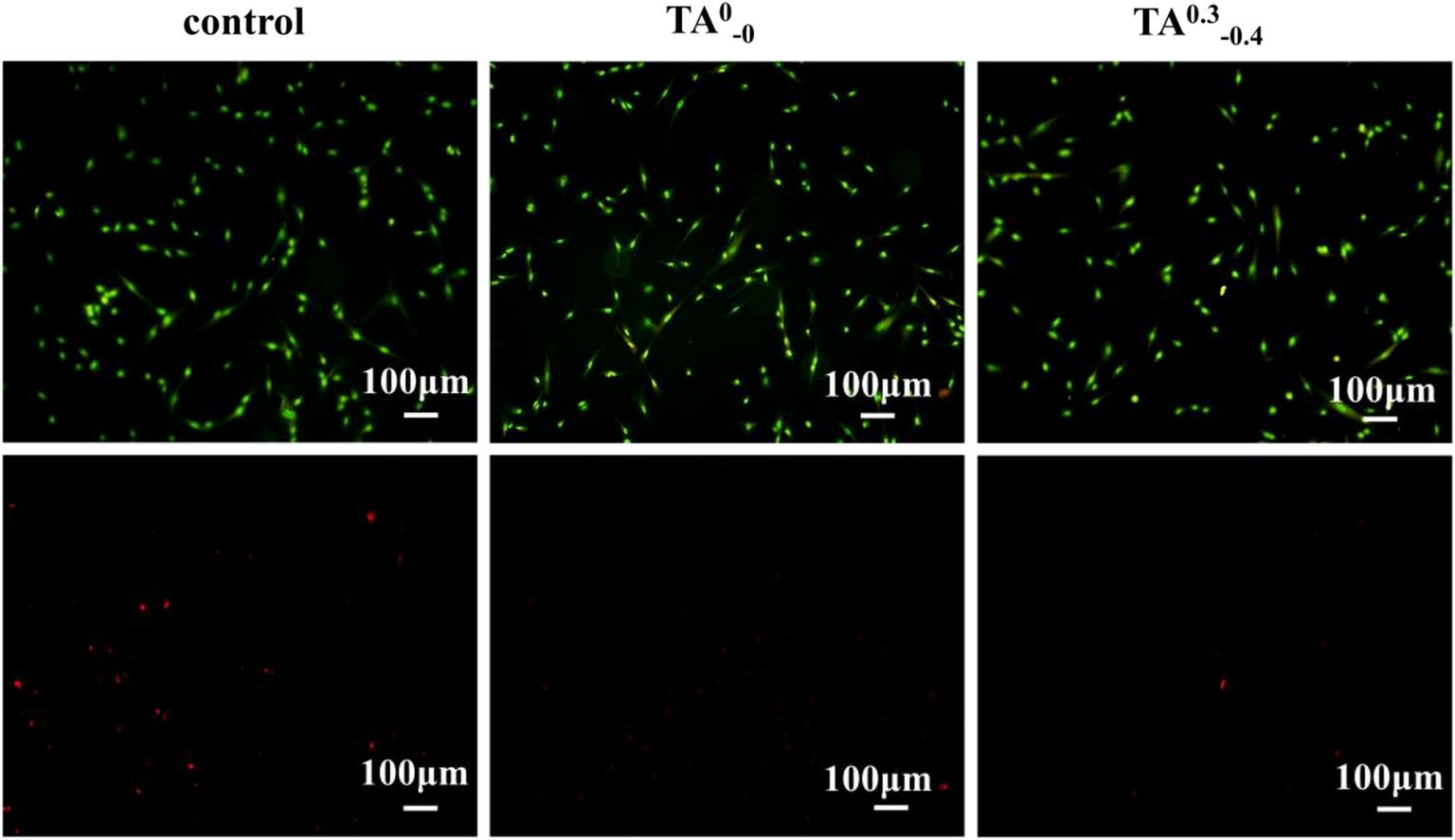
Figure of cell live/dead staining of 1 mg/mL hydrogel extracts.

The cytocompatibility of hydrogel dressings can be visually demonstrated by cell death staining. As shown in Fig. 15, where 1mg/mL hydrogel dressing extract was cultured with cells for 24 h, the cells remained active with almost no dead cells. The results indicated that the addition of tannic acid did not cause cell death, and the hydrogel dressing had good cell compatibility.

## Conclusion

In this experimental study, the mechanical properties of K/SA/CCS hydrogel were significantly improved by introducing a large amount of tannic acid modification so that it has a certain viscoelasticity and good toughness. In addition, we were surprised to find that hydrogels have good mechanical properties and excellent swelling behavior. According to the FT-IR and SEM analysis results, we speculated that a large amount of tannic acid formed hydrogen bonds with keratin, carboxymethyl chitosan, and sodium alginate, which improved its mechanical properties. However, the TA benzene ring contains a large number of phenolic hydroxyl groups, which are prone to overlapping electron clouds, and the repulsive effect of positive and negative charges leads to the increase of hydrogel pores. Consequently, taking into account the excellent swelling rate. Therefore, the composite hydrogel not only has mechanical properties that match the skin, but also provides a moist environment for wound healing and has great potential in supporting drug carrier transport. The hydrogel also has good antimicrobial and antioxidant properties as well as biocompatibility, which benefit wound healing and prevent infection. However, this study did not separately investigate the effects of keratin, sodium alginate, and carboxymethyl chitosan on the hydrogel properties, which interested scholars can further explore, providing new ideas for keratin hydrogel modification.

## Materials and methods

### Materials

Keratin was obtained by extraction from human hair and the method of extraction was in the support material. Carboxymethyl chitosan (carboxylation degree ≥ 80%, Shanghai Yuanye Biological Co., China), sodium alginate (analytically pure grade, Shanghai Aladdin Biochemical Technology Co., China), and tannic acid (pure grade, Beekman Biotechnology Co., China) were used by conducting experiments.

### Preparation of hydrogel

**Figure 16:**
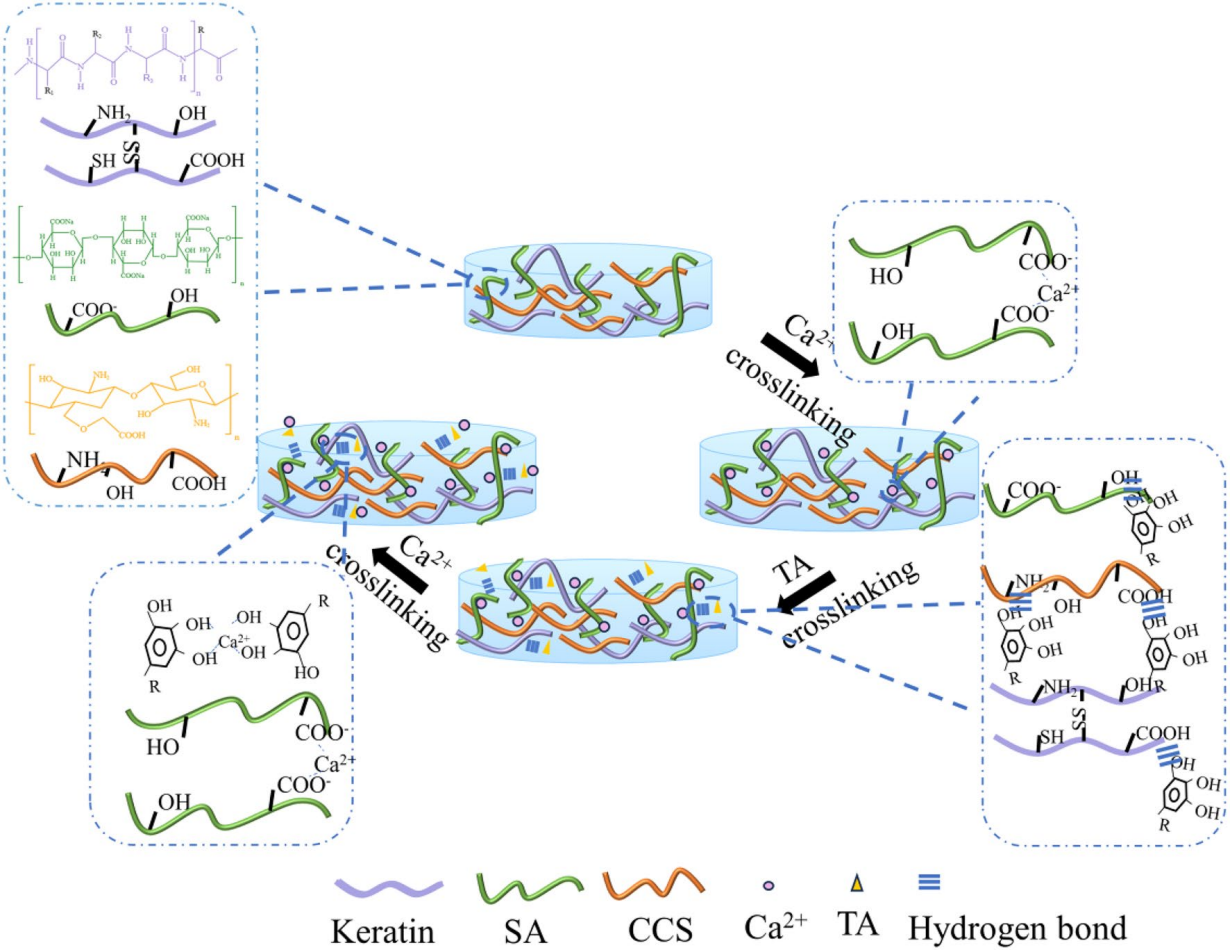
The schematic diagram of K/CCS/SA-TA/Ca^2+^ hydrogels.

CCS solution (1% w/v, glycerol/water = 1:1) was prepared first at 70 °C and dissolved with stirring, then SA (3% w/v) was added and further dissolved, then Keratin (1% w/v) was added at 45 °C and mixed with stirring. The mixture was transferred to a mold and left to stand overnight. Subsequently, the hydrogel was briefly immersed in Ca^2+^ solution and rinsed with deionized water. Then, the hydrogel was immersed in TA solution for 24 h. After washing with deionized water, the hydrogel was transferred to a calcium ionic solution for 1.5 h. This resulted in TA crosslinked modified K/SA/CCS composite hydrogel. TA^a^_−b_ denotes: a is the TA content a w/v, and b is the glycerol content b v/v. Ca^2+^_−c_: c is the Ca^2+^ content c w/v in CaCl_2_, solution and glycerol: water = 1:4 v/v in solution. The formation of the hydrogel is shown schematically in Fig. 16.

### Characterization

To explore the synthesis of TA-modified K/CCS/SA hydrogels and the interactions between the chemical groups, the FT-IR spectra of the materials were tested by using Nicolet iS 10 Fourier IR spectrometer (USA). Keratin, CCS and SA powders, K/CCS/SA hydrogels, and TA-modified K/CCS/SA hydrogel dressings were lyophilized in the LGJ-H20 lyophilizer (Beijing Sihuan Freeze-drying Co., Ltd. (China), and then, powdered with a mortar and pestle for FT-IR spectroscopy. The FT-IR spectra were scanned in the 500–4000 cm^−1^ range under vacuum.

### Mechanical properties

The tensile properties of the hydrogel samples were determined using the universal testing systems (Instron 6800 Series, UK) at room temperature. Each test sample was 10 mm in diameter, 50 mm in length, and 20 mm/min in tensile speed, with three parallel pieces in each group.

### Water retention test

The hydrogels were placed in a constant temperature incubator (25 °C), the initial mass was calculated, and they were weighed at regular intervals to calculate the water retention using the Eq. (1). Besides, the tensile states of TA^0.3^_−0.2_ and TA^0.3^_−0.4_ at the initial time and after 24 h at 25 °C were compared by a universal testing systems.

1 $$WR=\frac{{M}_{t}}{{M}_{0}}\times 100\%$$

*M*_0_ is the initial weight of hydrogels (g),* M*_*t*_ is the weight of hydrogels after t d (g).

### Anti-freezing test

Anti-freezing experiment was performed by placing the hydrogel samples in − 20 °C environment, and after 24 h, their tensile properties were examined and compared with the initial state.

### Rheological properties test

The energy storage modulus and loss modulus of hydrogels were tested by rheometer (MCR 102e, Austria), and the prepared hydrogel samples were tested at 25 °C. Dynamic scanning at 1% strain with angular velocities ranging from 0.01 to 100 Hz, was performed to detect the relationship between the energy storage modulus and loss modulus and frequency.

### Swelling ability

To assess the water-absorbing swelling property of hydrogels, the swelling ratio of hydrogel dressings was determined using the weighing method. The hydrogel was first freeze-dried, and then a sample was weighed before being soaked in PBS (pH 7.4) solution. The hydrogel dressing sample was periodically removed from a constant temperature incubator set at 37 °C, and any excess water on the surface was carefully eliminated using filter paper. Subsequently, the hydrogel samples were weighed again, and the mass of the sample was recorded. This procedure was repeated until the hydrogel samples reached swelling equilibrium in the PBS solution. Each representative group consisted of three parallel pieces. The swelling rate was calculated using the following Eq. (2) :

2 $$SR=\frac{\left({W}_{t}-{W}_{0}\right)}{{W}_{0}}$$

*W*_0_ is mass after lyophilization (g), *W*_*t*_ is mass after water absorption (g).

### SEM

The geometry and morphology of the hydrogels were observed by the camera and scanning electron microscopy (SEM JSM-7800F, Japan), respectively. Firstly, the hydrogel samples were freeze-dried. Subsequently, the hydrogel samples were prepared by vacuum deposition of a gold layer, which were then placed on a conductive adhesive tape and SEM images were taken at an accelerating voltage of 5 kV.

### Antibacterial studies

The fabricated hydrogels exhibited antibacterial potential which was assessed by taking bacterial *S. aureus* and *E. coli*. by disc diffusion method^41^. The prepared TA^0^_−0_, TA^0.1^_−0.4_, TA^0.3^_−0.4_, and TA^0.5^_−0.4_ hydrogel samples were sterilized under UV irradiation for 6 h. Bacteriostatic inhibition experiments were performed with *S. aureus* and *E. coli.* Firstly, the bacteria were activated in liquid LB medium at 37 °C, 180 rpm for 12 h. Then, the OD value was determined by enzyme marker, and the colonies of *S. aureus* cultured in liquid LB medium were diluted to 10^7^ CFU/mL, and colonies of *E. coli* were diluted to 10^6^ CFU/mL. Subsequently, the appropriate bacterial suspension was spread evenly on a solid culture plate, and then the sterilized hydrogel was carefully placed on the plate for incubation at 37 °C up to 12 h. Further, the diameter of the zone of inhibition was measured to find the antibacterial efficiency.

### DPPH assay

The antioxidant efficiency of hydrogel dressings was evaluated using scavenging of stabilized 1,1-diphenyl-2-pyridylhydrazine (DPPH) radicals was determined according to the procedure of Siripatrawan and Hartewith^42^ with minor modifications. Briefly, each composite hydrogel sample (15 mg) was ground into powder form and added with 3 mL of freshly prepared DPPH solution (0.1 mM) in 95% ethanol. The scavenging activity was evaluated by monitoring the absorbance decrease at 517 nm after storing the sample in the dark for 30 min. The DPPH radical scavenging activity was calculated by using the following Eq. (3):

3 $${DPPH}_{clearance}=\frac{{A}_{blank}-{A}_{sample}}{{A}_{sample}}\times 100\%$$

In the above equation, A_*blank*_ is the DPPH uptake of the blank (DPPH + ethanol); A_*sample*_ is the DPPH uptake of the hydrogel moiety (DPPH + ethanol + hydrogel).

### Hemolysis evaluation

The hemocompatibility of the prepared hydrogels was determined by hemolysis test according to previous reports^43^. Firstly, 4 mL blood samples were mixed with 1 mL 3.8 w% Na_3_Cit solution to prevent blood clotting. Subsequently, 5 mL treated blood was diluted with 5 mL saline solution for further usage. The sample extract solution was mixed with rabbit hemocyte suspension in the ratio of 10/0.2, followed by incubating at 37 °C for 1 h. Then, the hemocyte suspensions were centrifuged at 1000 rpm for 5 min, and supernatant was detected on an enzyme labeling apparatus at 540 nm (OD_540_). Distilled water was employed as a positive control, while normal saline was employed as a negative control. Three parallel samples per group. The hemolytic ratio was calculated according to the following Eq. (4):

4 $${Hemolytic}_{ratio}=\frac{{OD}_{samples}-{OD}_{nc}}{{OD}_{pc}-{OD}_{nc}}\times 100\%$$

In the above equation, OD_*samples*_ is the absorbance of the hydrogel samples, OD_*nc*_ is the absorbance of the negative control and OD_*pc*_ is the absorbance of the positive control.

### Cell viability test in vitro

The cytotoxicity of the hydrogel samples was studied via an extract test using BEAS-2B as model cells. For this purpose, the previously sterilized hydrogel samples were incubated with PBS buffer solution at room temperature, and hydrogel extract were collected after 24 h. BEAS-2B cells were cultured in the medium under standard culture conditions (37 °C, 5% CO_2_) until the cell density reached 80–90%. Subsequently, BEAS-2B cell suspension with a density of 10^3^–10^4^ to each hole, 100 μL per well, was uniformly pipetted into 96-well plates. The experimental group treated the cells with different concentrations (0.5 mg/mL, 1 mg/mL, 1.5 mg/mL) of hydrogel extracts, three wells for each concentration, and the control group was an equal volume of PBS buffer solution without hydrogel. The cells continued to be cultured under the same conditions for 24 h. An inverted microscope observed the cell morphology. 5 mg/mL of 20 μL of MTT solution was added to each well, and the incubation was continued for 4 h. The incubation was terminated, and the culture solution was carefully discarded. After the reduction of MTT into formazan salt by viable cells, 150 μL Dimethyl sulfoxide was added to dissolve the formazan salts. The absorbance of each well was measured by using an enzyme marker at OD_490_, and the cell survival rate was calculated according to the following formula:

5 $$CV(\%)=\frac{{A}_{s}-{A}_{b}}{{A}_{c}-{A}_{b}}\times 100\%$$

In the above formula, A_s_ is the absorbance of the experimental group, A_b_ is the absorbance of the blank group (containing only culture solution and MTT), and A_c_ is the absorbance of the control group.

Cell viability staining assay: Cells were cultured according to the steps of the in vitro cell toxicity assay and hydrogel extracts of 1 mg/mL of them were subjected to Calcein-AM/PI live-dead staining method for macroscopic evaluation of cell proliferation. First, the staining working solution was configured and set aside. After the cells were incubated with the hydrogel extract for 24 h, the medium was aspirated and washed carefully with a sterile PBS solution. Under the condition of light protection, 100 μL of live-dead staining working solution was added to each well, incubated in 37 °C, 5% CO_2_ incubator for 30 min, and then observed and photographed using an inverted fluorescence microscope.

### Ethics statements

This study was approved by the Ethics Committee of the Chinese People's Liberation Army Army Special Medical Center, and the experimental protocol was carried out in accordance with the guidelines for exemption from ethical review. The person who took the sample obtained and signed a written informed consent form from the donor.

### Supplementary Information

Supplementary Information.

## Data Availability

Data is provided within the manuscript and supplementary information files.
