# Supplementary material for: Tannic acid modified keratin/sodium alginate/carboxymethyl chitosan biocomposite hydrogels with good mechanical properties and swelling behavior
Source: Sci Rep. 2024 Jun 4;14:12864. doi: 10.1038/s41598-024-63186-6 (PMC11150462; doi:10.1038/s41598-024-63186-6)
Supplement: Supplementary file 1 — Supplementary Information. [file 41598_2024_63186_MOESM1_ESM.docx]

Tannic acid modified keratin/sodium alginate/carboxymethyl chitosan biocomposite hydrogels with good mechanical properties and swelling behavior

**Liqing Zhu^1^, Fenfen Ouyang^1^, Xue Fu^1^, Yimei Wang^1^, Ting Li^1^, Min Wen^1^, Guodong Zha^3^, Xue Yang^2^***

**1** College of Chemistry and Chemical Engineering, Chongqing University of Science and Technology, Chongqing, China. **2** Department of Pharmacy, Army Medical Center of PLA, Chongqing, China.**3** HEMOS (Chongqing) Bioscience Co., Ltd. Building #2, No.216, Jianshan Road, Bishan District, Chongqing, China.

* Corresponding author

E-mail: yangxue198902@163.com (XY)

# Supporting information

Keratin was extracted from barbershops of discarded human hair according to a modified by Bochu Wang’s lab^1^: The human hair was firstly washed with the 0.5% SDS (w/v) to remove the surface grease and 60℃ dried overnight. After then, 0.5 M Thioglycolic acid was considered reductant to break cystine bonds adjusted to pH 11.0 for 15 h, and the reduction solution was retained through the filtration. The crude fraction of keratin was extracted using a 100 mM Tris base solution for 2 h, followed by a second extraction using deionized (DI) water. The extractions were combined and centrifuged at 8,000 rpm for 10 minutes at 4°C. In addition, the extraction’s pH was adjusted to 7.4, and the next step involved dialysis using a dialysis bag (MW3500) for 3 d. The resulting extracts were freeze-dried and stored.

# Results and discussion

Electrophoretic separation plots (S1 Fig) of human hair keratin extracts reveal multiple bands ranging from 10-65 kDa. In line with previous studies on human hair keratin extracts, the proteins observed at approximately 45 kDa are classified as type I keratins. At the same time, those at around 55 kDa are categorized as type II keratins.

The FT-IR spectrum of Keratin indicates several critical peaks and their corresponding vibrations. As shown in S2 Fig, the similar typical characteristic at 3405 cm^-1^ corresponding to t the stretching vibration of -OH and -NH_2_ groups. The stretching vibrations of the C=O stretching (amide I), N-H stretching (amide II) and C-N bending (amide III) were also found at 1654 cm^-1^, 1541 cm^-1^ and 1234 cm^-1^, respectively, which confirmed that amide bonds exist in the keratin. The band observed at 2963 cm^-1^ corresponds to the stretching vibration of C-H bonds. Additionally, a distinctive absorption peak at 609 cm^-1^ indicates the presence of S-S groups.

As shown in S3 Fig, the UV full-wave scanning absorption peak of keratin is around 275 nm because the presence of tryptophan, cystine, tyrosine, and histidine in keratin gives it UV absorption properties.

In the XRD pattern of keratin (S4 Fig), there is a faint diffraction peak observed at 2θ=10.3°, indicating the presence of its α-helical and β-folded structure. Additionally, a broader diffraction peak is observed at 2θ=21.2°, corresponding to its β-folded system. However, this peak exhibits a lower intensity and more overall width, suggesting that the keratin powder possesses an amorphous structure.

# Experimental

Sodium dodecyl-sulfate polyacrylamide gel electrophoresis (SDS-PAGE) analysis: The SDS-PAGE procedure described by was followed to separate and visualize the keratin proteins. The lyophilized keratin powders were dissolved in ultrapure water. The solution was mixed with 4 × Loading buffer. The mixture was heated at 100°C for 5 minutes to prepare the sample for SDS-PAGE. The denatured solution was loaded onto precast 5%-10% gradient Tris-HCl gels. Electrophoresis was conducted at 80 V for 1 hour, followed by 110 V for 2 h. After electrophoresis, the gels were stained with 0.02% (w) Coomassie Brilliant Blue G-250 for 2 h and subsequently destained with acetic acid twice.

Fourier transforms infrared spectroscopy analysis: The chemical structure of keratin was analyzed by a FT-IR (Nicolet iS 10, USA), specimens were previously prepared potassium bromide (KBr) method at wave number range from 4000 to 500 cm^-1^.

Ultraviolet-visible (T9, China) spectral analysis: Keratin was dissolved in water, passed through a 200 mesh sieve, and its absorption spectrum was measured using a UV spectrophotometer within the 220-400 nm range.

X-ray diffraction analysis: The Freeze-dried keratin were characterized by X-ray diffraction (SmartLab-9, Japan). Data were obtained in the range from 5°to 45°(2θ) at 5°/min with a step of 0.02°.

# Data availability

Data is provided within the manuscript and supplementary information files.

# References

1. Luo, T., Hao, S., Chen, X., Wang, J., Yang, Q., Wang, Y., Weng, Y., Wei, H., Zhou, J., and Wang, B., *Materials Science and Engineering: C*., 2016, vol. 63. pp. 352-358. https://doi.org/10.1016/j.msec.2016.03.007.

# Funding

This study supported by Chongqing Clinical Pharmacy Key Specialties Construction Project and HEMOS (Chongqing) Bioscience Co., Ltd.

# Conflict of interest

The authors declare no competing interests.

# Author Contributions

Conceptualization, Data curation and Formal analysis, F. O., Funding acquisition, X.Y. L.Z., and G.Z., Investigation, Y.W., T. L. and M.W. Supervision, L.Z., X.Y. and X.F. Writing - original draft, F.O., Writing - review & editing, L.Z., X.Y. X.F. and G.Z., All authors read and approved the manuscript.

# Ethics statements

This study was approved by the Ethics Committee of the Chinese People's Liberation Army Army Special Medical Center, and the experimental protocol was carried out in accordance with the guidelines for exemption from ethical review. The person who took the sample obtained and signed a written informed consent form from the donor.

**S1 Fig. Extracted keratin SDS-PAGE graph.**

**S2 Fig. Extracted keratin FT-IR graph.**

**S3 Fig. Extracted keratin UV-vis graph.**

**S4 Fig. Extracted keratin XRD graph.**


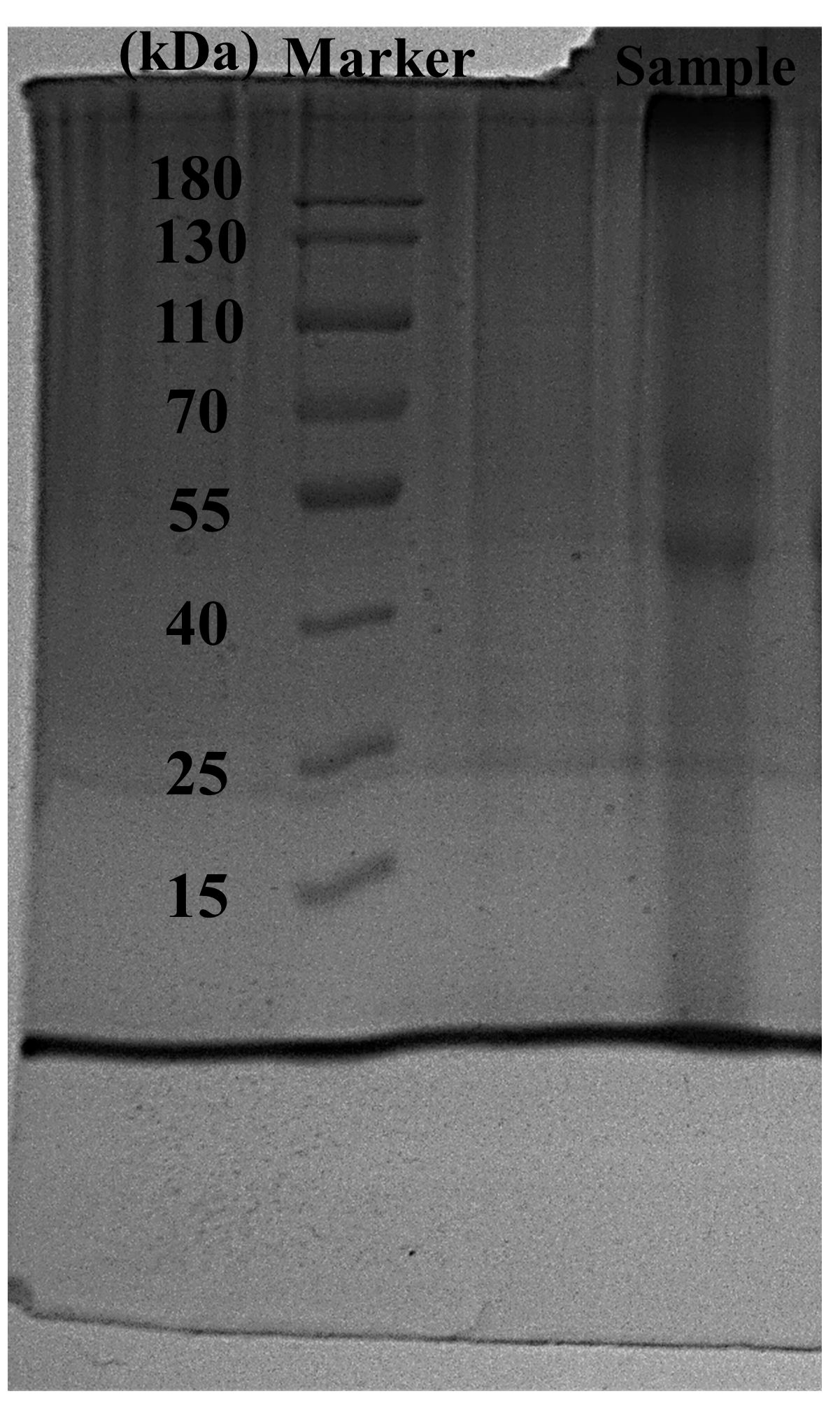


S1 Fig.





S2 Fig.





S3 Fig.





S4 Fig.
